# Recurrence risk stratification for locally advanced cervical cancer using multi-modality transformer network

**DOI:** 10.3389/fonc.2023.1100087

**Published:** 2023-02-16

**Authors:** Jian Wang, Yixiao Mao, Xinna Gao, Yu Zhang

**Affiliations:** ^1^ School of Biomedical Engineering, Southern Medical University, Guangzhou, Guangdong, China; ^2^ Guangdong Provincial Key Laboratory of Medical Image Processing, Southern Medical University, Guangzhou, Guangdong, China; ^3^ Department of Radiation Oncology, Southern Medical University Nanfang Hospital, Guangzhou, Guangdong, China

**Keywords:** cervical cancer, recurrence risk stratification, multi-modality data, deep learning, transformer network

## Abstract

**Objectives:**

Recurrence risk evaluation is clinically significant for patients with locally advanced cervical cancer (LACC). We investigated the ability of transformer network in recurrence risk stratification of LACC based on computed tomography (CT) and magnetic resonance (MR) images.

**Methods:**

A total of 104 patients with pathologically diagnosed LACC between July 2017 and December 2021 were enrolled in this study. All patients underwent CT and MR scanning, and their recurrence status was identified by the biopsy. We randomly divided patients into training cohort (48 cases, non-recurrence: recurrence = 37: 11), validation cohort (21 cases, non-recurrence: recurrence = 16: 5), and testing cohort (35 cases, non-recurrence: recurrence = 27: 8), upon which we extracted 1989, 882 and 315 patches for model's development, validation and evaluation, respectively. The transformer network consisted of three modality fusion modules to extract multi-modality and multi-scale information, and a fully-connected module to perform recurrence risk prediction. The model's prediction performance was assessed by six metrics, including the area under the receiver operating characteristic curve (AUC), accuracy, f1-score, sensitivity, specificity and precision. Univariate analysis with F-test and T-test were conducted for statistical analysis.

**Results:**

The proposed transformer network is superior to conventional radiomics methods and other deep learning networks in both training, validation and testing cohorts. Particularly, in testing cohort, the transformer network achieved the highest AUC of 0.819 ± 0.038, while four conventional radiomics methods and two deep learning networks got the AUCs of 0.680 ± 0.050, 0.720 ± 0.068, 0.777 ± 0.048, 0.691 ± 0.103, 0.743 ± 0.022 and 0.733 ± 0.027, respectively.

**Conclusions:**

The multi-modality transformer network showed promising performance in recurrence risk stratification of LACC and may be used as an effective tool to help clinicians make clinical decisions.

## 1 Introduction

Cervical cancer is one of the most common malignancies in females worldwide, which ranks as the 4th leading cause of death among cancers in women (1). Locally advanced cervical cancer (LACC), as the cervical cancer in IB2, IIA2 and IIB~IVA stages, is generally considered as a local mass with the size larger than 4cm or invades the surrounding tissues, in which distant metastasis does not occur (1). In clinical practices, the treatment for patients with LACC does not follow the same pattern (2). Most LACC patients are routinely treated with concurrent chemoradiation therapy, and the prognosis is heterogeneous (3). Despite neoadjuvant and adjuvant therapies are being tentatively introduced into the treatment regimen, the overall outcomes are not significantly improved (4, 5). The potential reason may be associated with the small-scale cohorts benefited from the neoadjuvant and adjuvant treatments, and all of these patients are from the high-risk recurrence group (6). Therefore, an interesting and crucial topic is to accurately predict recurrence risk so as to formulate the individualized therapeutic schedule for LACC patients.

With the rapid development of imaging techniques, imaging examinations has been considered as a routine for patients with cervical cancer. Currently, several studies have conducted recurrence and prognosis analysis for cervical cancer by extracting and evaluating high-throughput imaging features (7, 8). For example, some work has carried out texture analysis based on positron emission tomography (PET) or magnetic resonance (MR) images to predict the recurrence risk of cervical cancer (9, 10). In addition, the ultrasound (US) and computed tomography (CT) images were also used in recurrence-related tasks, such as lymph node metastasis prediction and survival assessment (11, 12). However, few studies have tried to focus on the recurrence risk stratification of LACC. Moreover, previous methods only utilized the information from mono-modality data and did not take multi-modality complementary information into consideration. Consequently, it is desirable to design an efficient model to make full use of multi-modality data (*i.e.*, CT and MR images) for accurately stratifying the recurrence status of LACC.

In recent years, deep learning has demonstrated its superiority over conventional radiomics methods based on hand-crafted features (13), and it avoids the complex hand-crafted feature extraction (14). Transformer, as one of the most popular deep learning architectures, has been successfully applied to various medical image analysis tasks and shows promising performance (15–17). In this study, we investigated the ability of transformer network in recurrence risk stratification of LACC by using non-contrast enhanced CT images and T1-Weighted MR images. Specifically, the transformer network consisted of three modality fusion modules to extract multi-modality and multi-scale information, and a fully-connected module to perform recurrence risk prediction. The performance of the model was assessed by six metrics. The results showed that our proposed model significantly outperformed the conventional radiomics methods.

## 2 Materials and methods

### 2.1 Patients

This study was approved by the Institutional Review Board, and written informed consent requirement was waived. Totally, 104 patients with pathologically diagnosed LACC between July 2017 and December 2021 were retrospectively enrolled. For all participants, the inclusion criteria were as follows: (1) patients who pathologically confirmed LACC; (2) patients who underwent radiotherapy as the main treatment; (3) patients who underwent both CT and MR examinations within three weeks before radiotherapy. The exclusion criteria were as follows: (1) external irradiation treatment was interrupted for more than one month; (2) the radiation dose to tumor was less than 80Gy; (3) surgery was performed before radiotherapy. All enrolled participants with matched multi-modality data were randomly divided into training and testing cohorts at a ratio of 2: 1 to develop and assess the network, respectively.

Recurrent tumors were classified into local, regional, or distant progressive tumors after concurrent chemoradiotherapy was completed. Clinical follow-up exams of the patients were performed every 3 months until 36 months. Physical examination and tumor markers were checked. Imaging examination of pelvic MRI (CT for special patients) was performed when suspected of recurrence and the biopsy was performed for confirmation.

The clinicopathologic data of all enrolled patients, including age, tumor stage (FIGO 2009^1^), pathologic diagnosis, lymph node status and dose of radiotherapy, were obtained from medical records for statistical analysis and the recurrence status of all patients was also followed up.

### 2.2 CT and MR image acquisition

The CT images were collected from the CT scanner (Philips Healthcare, Best, The Netherlands). The scanning current and voltage were 300 mAs and 120 kV, respectively. Both slice thickness and slice distance were set to 3 mm, and the resolution was 512×512 pixels. The scanning range of CT was from the diaphragm to the proximal femur. The MR images were acquired from four MR scanners: an Achieva 3T MR scanner (Philips Medical Systems, Best, The Netherlands), with the repetition time of 431.5-697.4 msec, echo time of 10 msec, slice thickness of 5 mm, flip angle of 90°, percentage phase field of view of 100%, and matrix of 320×320 or 560×560; an Ingenia 3T MR scanner (Philips, Best, The Netherlands), with the repetition time of 431.5-697.4 msec, echo time of 10 msec, slice thickness of 5 mm, flip angle of 90°, percentage phase field of view of 100%, and matrix of 320×320 or 560×560; a Signa HDxt 1.5T MR scanner (GE Medical Systems, Milwaukee, Wis, USA), with the repetition time of 200-620 msec, echo time of 8.104 msec, slice thickness of 6 mm, flip angle of 90°, percentage phase field of view of 100%, and matrix of 512×512; an OPTIMA MR360 1.5T MR scanner (GE Healthcare, Milwaukee, Wis, USA), with the repetition time of 393-1179 msec, echo time of 12.36 msec, slice thickness of 5-7.5 mm, flip angle of 90°, percentage phase field of view of 100%, and matrix of 512×512. The scanning range of MR scanners was the whole pelvic area.

### 2.3 Imaging registration and VOI segmentation

In this study, we mainly focused on the imaging information of the primary tumor regions for recurrence risk stratification. The lymph node status was not included in the model. The specific reason is that the patients included in this study were all patients who had not undergone surgery, and there was no gold standard (pathological result) to verify the presence of lymph node metastasis. Previous studies (18, 19) have also shown that it is sufficient to use only the imaging information of primary lesions for cancer prognosis analysis, and the method selection of this study is generally in line with the previous research norms.

**Figure 1:**
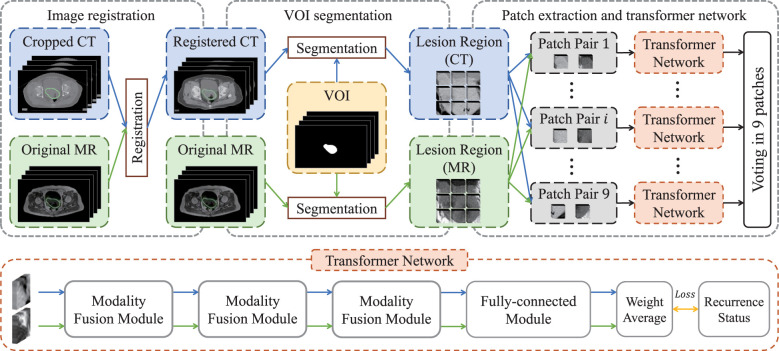
The flow chart of the proposed model for the recurrence risk stratification of LACC.

We chose non-contrast enhanced CT and T1-weighted MR images to carry out imaging analysis and used T1-weighted MR to contour the tumor. The main reason is that MR imaging has higher soft-tissue contrast resolution, so cervical cancer, which originates in the pelvis and is mixed with surrounding soft tissues, can be well identified. In order to ensure that the primary lesion area can be accurately located in CT images, we registered them with the MR images and then used the VOIs (*i.e.*, primary tumor regions) of MR images to extract lesion regions in both registered CT images and original MR images. Specifically, as shown in **Figure 1**, we first cropped CT images to focus on the pelvic area, and then aligned cropped CT images to the MR images via elastic registration (3D Slicer software 4.11). The VOIs were manually delineated on T1-weighted MR images by using ITK-SNAP 3.6 (ITK-SNAP 3.x Team, www.itksnap.org) by a radiologist with 10 years of experience.

### 2.4 Patch extraction

The lesion regions of all patients were resampled into a volume with the specified resolution of 86×86×12, and then zero-mean normalization was applied to each volume for image standardization so as to eliminate the bias introduced by inconsistent imaging parameters (20). Subsequently, each volume was split into nine patches with the size of 32×32×12, in which adjacent patches had 5-voxel overlap alone coronal and sagittal directions. Finally, paired multi-modality patches were taken as the input of transformer network for recurrence risk prediction.

### 2.5 Transformer network

**Figure 2:**
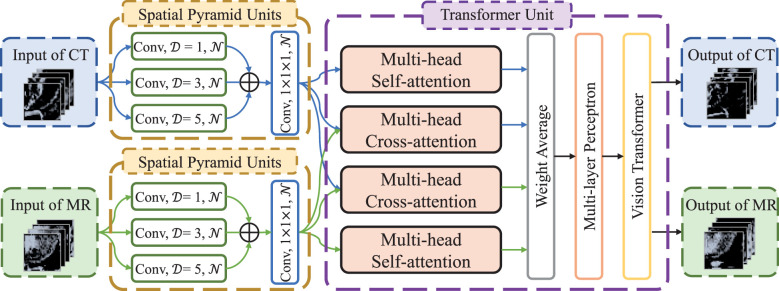
The architecture of modality fusion module. *D* is dilation rate in the convolutional layers and *N* is the number of convolutional kernels, which is set to 4, 8 and 16, respectively, in the three modality fusion modules.

The flow chart of transformer network is shown in **Figure 1**. The transformer network was composed of three modality fusion modules and a fully-connected module. The modality fusion module (as shown in **Figure 2**) consisted of two spatial pyramid units and a transformer unit. The former was used to extract the multi-scale image features effectively. The spatial pyramid features were obtained by utilizing three paralleled 3×3×3 convolutional layers with the dilation rates of 1, 3 and 5, respectively. Then, a pixel-wise summation operator and a 1×1×1 convolution layer were used to aggregate these features. In order to avoid gradient vanishing and accelerate convergence, a batch normalization (BN) layer and a Leaky ReLU nonlinearity operation were plugged after each convolutional layer. Subsequently, a transformer unit was utilized to capture semantic features between two modalities data (21). Specifically, we performed two multi-head self-attention operations for each modality to learn modality-specific information, and two multi-head cross-attention operations to extract complementary features from the other modality. Afterwards, weight average operator was adopted to aggregate all feature maps, and the weights of different features were learned automatically. A multi-layer perceptron (MLP) layer and a vision transformer (ViT) unit (17) were then applied to further extract semantic representations. Subsequently, CT and MR features were fed into the fully-connected module that contained a global average pooling layer, three stacked fully-connected layers (with the node number of 8, 4, 1, respectively) and a Sigmoid activation function to generate the patch-level predictions for CT and MR images, respectively. Another weight average operation was then used to aggregate the predicted probabilities of two modalities. Finally, we adopted the voting strategy to integrate the predicted probabilities of nine paired patches to obtain patient-level recurrence risk prediction.

### 2.6 Conventional radiomics methods and deep neural networks

To verify the effectiveness of our method, we compared the proposed method with some conventional radiomics methods and deep neural networks. For conventional radiomics methods, followed by (22), we extracted 4 non-texture features (including volume, size, solidity and eccentricity) and 10320 texture features from each modality for each patient. Subsequently, we utilized a filter-based feature selection method, namely Relief algorithm (23), to select the features with the best distinguishing power. The selected features were then used to construct the decision tree classifier (24), naive bayes classifier (25), k-nearest neighbor (KNN) classifier (26) and support vector machine (SVM) classifier (27), respectively, for recurrence risk prediction. For comparison with deep neural networks, we reproduced ResNet18 (28) and MobileNetV1 (29) networks. We employ the same data preprocessing strategy as the proposed method, and then utilized the input-level fusion strategy to fuse multi-modality images into deep networks by multi-channel.

### 2.7 Implementation details and statistical analysis

We conducted data augmentation strategy (*i.e.*, random affine transformation) to generate sufficient images to train the transformer network so as to alleviate the overfitting and data imbalance issues (30). Specifically, all VOIs were first scaled to the volume with the size of 560×560×20 and then underwent rotation (within π/18, π/18, π/4 in the coronal, sagittal and transverse sections, respectively) and zoom (between 0.75 and 1.25) operations, followed by patch extraction. For each method, we randomly divided the training and validation sets five times to verify the robustness of the method. In the training stage, we utilized binary cross entropy as the loss function and recurrence status as the label. And Kaiming initialization (31) and Adam optimizer (32) were adopted to initialize and optimize model's parameters. The model was complemented under the PyTorch (version 1.10.1) based on Python (version 3.8.0). All intensive calculations were offloaded to a workstation with Central Processing Unit (CPU) of Intel(R) Xeon(R) CPU E5-2623 v3 @ 3.00GHz, Graphics Processing Unit (GPU) of NVIDIA Pascal Titan X, and 125 GB RAM. The conventional radiomics model was carried out by MATLAB software (version 2020a).

Continuous variables were expressed as means (standard deviation), and categorical data were expressed as numbers (percentage). The model's prediction performance was assessed by six metrics, including the area under the receiver operating characteristic curve (AUC), accuracy, f1-score, sensitivity, specificity and precision. Univariate analysis with F-test was conducted to compare differences between clinical variables and recurrence status of LACC, while T-test for the difference comparison of AUCs, and significant difference was defined by P < 0.05. All statistical analyses were implemented using R software (version 4.0.2).

## 3 Results

### 3.1 Clinical characteristics

**Figure 3:**
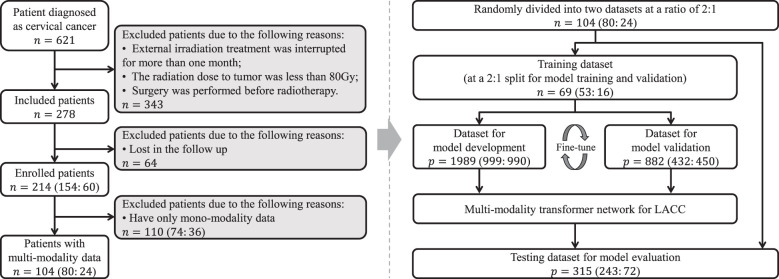
Patient inclusion and exclusion criteria, and the study flow chart.

The clinical baseline characteristics of the enrolled participants are shown in **Table 1**. The inclusion and exclusion criteria are shown in **Figure 3** (left). To develop and assess the proposed model, the enrolled patients were randomly divided into the training cohort and testing cohort with an approximate ratio of 2: 1. Then, in the training cohort, we further portioned two-thirds samples for training the network and the rest for validating the network, respectively. We performed three-fold augmentation for non-recurrence cases and ten-fold augmentation for recurrence cases in the training set to bridge the quantitative gap between two categories. Totally, 1989 (non-recurrence: recurrence = 999: 990), 882 (non-recurrence: recurrence = 432: 450) and 315 (non-recurrence: recurrence = 243: 72) patches were generated from training, validation and testing cohorts. The flow chart of the study is shown in **Figure 3** (right). The all cohorts maintained the same class distribution.

**Table 1:** Clinical characteristics of recurrence and non-recurrence cohorts.

	Total	Non-recurrence cohort	Recurrence cohort	P-value*
Number	N = 104	N = 80	N = 24	
Characteristics				
Age (year)	56.68 (8.88)	55.24 (7.89)	59.00 (10.12)	0.2023
FIGO^1^ (2009 stage)				0.0008
IB2	5	5 (100%)	0 (0%)	
IIA1	3	3 (100%)	0 (0%)	
IIA2	9	8 (89%)	1 (11%)	
IIB	46	37 (80%)	9 (20%)	
IIIA	7	6 (86%)	1 (14%)	
IIIB	30	21 (70%)	9 (30%)	
IVA	2	0 (0%)	2 (100%)	
IVB	1	0 (0%)	1 (100%)	
Unknown	1	0 (0%)	1 (100%)	
Pathologic diagnosis				0.9002
Squamous cell carcinoma	96	74 (77%)	22 (23%)	
Adenocarcinoma	4	3 (75%)	1 (25%)	
Unknown	4	3 (75%)	1 (25%)	
Lymph node status				0.2674
Pelvic + Retroperitoneal	33	24 (73%)	9 (27%)	
Pelvic + groin	2	1 (50%)	1 (50%)	
Pelvic	15	11 (73%)	4 (27%)	
Retroperitoneal	3	2 (67%)	1 (33%)	
Unknown	51	42 (82%)	9 (18%)	
Dose of radiotherapy				0.8471
45Gy/25F	9	7 (78%)	2 (22%)	
45-60Gy/25F	6	6 (100%)	0 (0%)	
50.4Gy/28F	2	2 (100%)	0 (0%)	
50.4-60Gy/28F	46	31 (67%)	15 (33%)	
Unknown	41	34 (83%)	7 (17%)	

^1^ FIGO, International Federation of Gynecology and Obstetrics.

* P-value is derived from the univariate analysis with F-test.

### 3.2 Training process and prediction performance of transformer network

**Figure 4:**
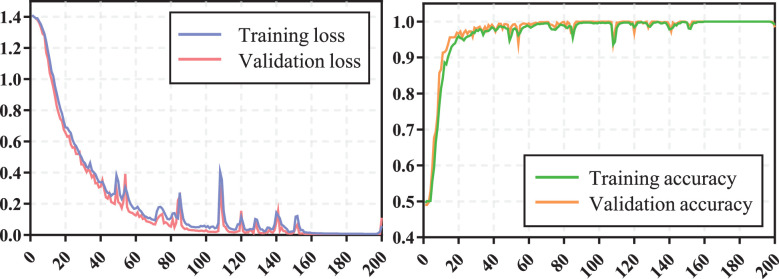
The training process of transformer network.

The training process of transformer network is shown in **Figure 4**, which suggests that the loss of model gradually converged and the accuracy gradually stabilized as iterations number increased. The prediction performance of transformer network on recurrence risk prediction of LACC is listed in **Table 2**. From **Table 2**, we can observe that the transformer network can accurately predict the recurrence status of all samples in the training cohort. Meanwhile, it achieved good performance with AUC of 0.819 ± 0.038, accuracy of 0.869 ± 0.023, f1-score of 0.914 ± 0.016, sensitivity of 0.911 ± 0.038, specificity of 0.725 ± 0.094 and precision of 0.919 ± 0.025 in the testing cohort.

**Table 2:** Comparison results of the proposed method and other competing methods.

		AUC	Accuracy	F1-score	Sensitivity	Specificity	Precision	P-value^*^
Proposed	Training	0.987±0.025	0.986±0.029	0.984±0.031	0.971±0.058	1.000±0.000	1.000±0.000	
	Validation	0.989±0.021	0.988±0.024	0.987±0.027	0.975±0.050	1.000±0.000	1.000±0.000	
	Testing	0.819±0.038	0.869±0.023	0.914±0.016	0.911±0.038	0.725±0.094	0.919±0.025	
Decision Tree	Training	0.968±0.017	0.957±0.016	0.972±0.010	0.974±0.009	0.900±0.075	0.970±0.022	4.20e-05
	Validation	0.953±0.053	0.943±0.036	0.962±0.023	0.950±0.025	0.920±0.098	0.975±0.031	
	Testing	0.680±0.050	0.703±0.034	0.787±0.026	0.711±0.028	0.675±0.061	0.881±0.023	
Bayes Classifier	Training	0.867±0.020	0.878±0.015	0.924±0.009	0.970±0.015	0.575±0.047	0.883±0.011	7.54e-05
	Validation	0.903±0.066	0.867±0.019	0.915±0.013	0.950±0.025	0.600±0.000	0.884±0.003	
	Testing	0.720±0.068	0.691±0.042	0.777±0.030	0.696±0.028	0.675±0.100	0.879±0.037	
KNN	Training	0.925±0.031	0.843±0.023	0.905±0.014	0.966±0.014	0.437±0.068	0.851±0.016	1.40e-03
	Validation	0.929±0.067	0.838±0.023	0.904±0.012	1.000±0.000	0.320±0.098	0.825±0.021	
	Testing	0.777±0.048	0.731±0.053	0.807±0.047	0.741±0.084	0.700±0.100	0.895±0.030	
SVM	Training	0.989±0.013	0.980±0.012	0.987±0.007	0.996±0.008	0.925±0.047	0.978±0.014	7.39e-07
	Validation	0.975±0.038	0.952±0.030	0.969±0.020	0.987±0.025	0.840±0.080	0.952±0.024	
	Testing	0.691±0.103	0.646±0.023	0.737±0.020	0.644±0.030	0.650±0.050	0.862±0.017	
MobileNetV1	Training	0.979±0.031	0.977±0.033	0.976±0.035	0.961±0.054	0.994±0.012	0.993±0.014	7.70e-03
	Validation	0.975±0.040	0.973±0.044	0.971±0.048	0.954±0.073	0.992±0.016	0.990±0.020	
	Testing	0.743±0.022	0.811±0.023	0.875±0.020	0.859±0.054	0.650±0.094	0.894±0.020	
ResNet18	Training	0.968±0.053	0.966±0.055	0.964±0.060	0.948±0.082	0.984±0.029	0.981±0.034	7.60e-03
	Validation	0.977±0.040	0.971±0.052	0.967±0.062	0.946±0.108	0.996±0.008	0.996±0.008	
	Testing	0.733±0.027	0.800±0.031	0.863±0.026	0.822±0.049	0.725±0.050	0.910±0.011	

* P-value is calculated by T-test to measure significant differences from proposed model.

### 3.3 Comparison with conventional radiomics methods and deep neural networks

**Figure 5:**
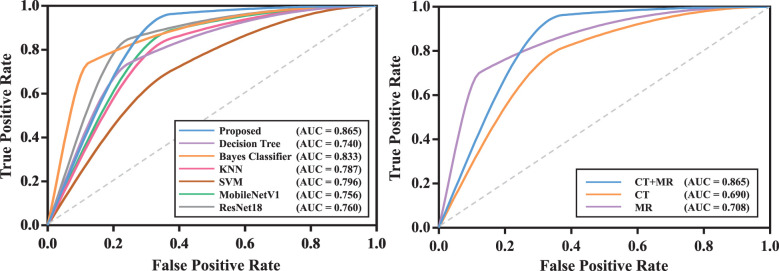
The ROC curves of models in testing cohort. The model with the best test result was shown. Left: ROC curves of all competing methods; Right: ROC curves of the proposed model with mono-modality and multi-modality data.

We compared the proposed transformer network with conventional radiomics methods and deep neural networks. The results are shown in **Table 2**. We can find that the transformer network is generally superior to other methods in both training, validation and testing cohorts. Particularly, in testing cohort, the transformer network achieved the highest AUC of 0.819 ± 0.038, while conventional radiomics methods got the AUCs of 0.680 ± 0.050, 0.720 ± 0.068, 0.777 ± 0.048 and 0.691 ± 0.103, respectively. The AUCs of the ResNet18 and MobileNetV1 were 0.743 ± 0.022 and 0.733 ± 0.027, respectively, which did not show competitive performances. We analyzed that these two classical networks both used the input-level modality fusion strategy, which made it difficult to establish the intrinsic relationship between different modalities of the same patient, resulting in the degradation of the model performance (15). By contrast, we adopted the transformer structure, and used its unique attention mechanism to fully learn the complementary information between modalities and mined discriminative semantic features. Therefore, the proposed model was more accurate and robust. **Figure 5** (left) plots the ROC curves of all competing methods in testing cohort.

### 3.4 Efficacy of multi-modality data

We compared the prediction performance of the proposed model on mono-modality data (*i.e.*, only trained with CT or MR images) and multi-modality data. The detailed experimental design can be found in **Supplementary Materials**. **Table 3** shows the experimental results, and **Figure S1** depicts the training process of transformer network on mono-modality data. We can see that the model with multi-modality data obtained the best results when compared with the models with only mono-modality data. **Figure 5** (right) exhibits the corresponding ROC curves, further validating the above-mentioned contents. It is not surprising about the observation, in that multi-modality data can provide more complementary information for the recurrence risk stratification of LACC.

**Table 3:** Comparison results of the proposed method on mono-modality data and multi-modality data.

		AUC	Accuracy	F1-score	Sensitivity	Specificity	Precision	P-value*
CT+MR	Training	0.987±0.025	0.986±0.029	0.984±0.031	0.971±0.058	1.000±0.000	1.000±0.000	
	Validation	0.989±0.021	0.988±0.024	0.987±0.027	0.975±0.050	1.000±0.000	1.000±0.000	
	Testing	0.819±0.038	0.869±0.023	0.914±0.016	0.911±0.038	0.725±0.094	0.919±0.025	
CT	Training	0.998±0.005	0.997±0.005	0.997±0.005	0.995±0.010	1.000±0.000	1.000±0.000	5.75e-04
	Validation	0.996±0.008	0.996±0.008	0.996±0.009	0.992±0.017	1.000±0.000	1.000±0.000	
	Testing	0.677±0.021	0.737±0.042	0.813±0.039	0.748±0.068	0.700±0.061	0.895±0.013	
MR	Training	0.996±0.008	0.996±0.009	0.995±0.009	0.991±0.018	1.000±0.000	1.000±0.000	2.82e-03
	Validation	0.996±0.008	0.996±0.008	0.996±0.009	0.992±0.017	1.000±0.000	1.000±0.000	
	Testing	0.676±0.068	0.720±0.066	0.795±0.049	0.704±0.052	0.775±0.146	0.914±0.055	

* P-value is calculated by T-test to measure significant differences from proposed method on multi-modality data.

### 3.5 Efficacy of key modules in transformer network

We also validated the efficacy of key modules in transformer network. The detailed experimental design and results can be found in **Supplementary Materials**.

## 4 Discussion

In this study, we developed and evaluated a transformer network for the recurrence risk stratification of locally advanced cervical cancer (LACC) based on computed tomography (CT) and magnetic resonance (MR) images. The proposed method achieved excellent prediction performance, which could be potentially used as an effective tool for the decision-making support in a non-invasive way.

The individualized treatment of cervical cancer is guided by the FIGO staging (33, 34). For patients with LACC, the preferred treatment is concurrent chemoradiation rather than surgery (3). However, unlike surgery treatment that can evaluate recurrence risk based on the resected tumor, the concurrent chemoradiation lacks of the conditions for adequate pathological evaluation after local biopsy. Hysteretic risk assessment and intervention would lead to cancer recurrence for partial patients. Therefore, it is desirable to accurately predict the recurrence risk of LACC so as to determine appropriate adjuvant treatment strategies.

Under the current advocacy of precision medicine (35) powered by patient data (36), personalized treatment is the inevitable trend of current medical technology development. The FIGO 2018 staging system has acknowledged the value of imaging for optimal risk stratification and treatment planning (37, 38) and European Society of Urogenital Radiology (ESUR) guidelines also affirmed the important role of MR images in the risk assessment of cervical cancer recurrence (39). Additionally, medical imaging acquisition and storage techniques enable the non-invasive analysis for various diseases, which efficiently assists clinicians in disease diagnosis, treatment and prognosis (40, 41). Typically, radiomics signatures have been widely used and show promising value (42, 43). With the widespread promotion of deep learning technology, the threshold for mastering such high-precision models has been completely lowered. Compared to conventional radiomics methods, deep learning simplifies the multi-step pipeline by automatically learning useful features from images, and exhibits better predictive performance (44). As one of the challenges of deep learning, large-scale data are needed for model training. However, the low incidence of LACC might lead to insufficient training data. To this end, in our work, we employed the patch-based strategy to extract a large amount of image patches from each patient and additionally performed data augmentation to scale up training data and prevent overfitting. Furthermore, we designed a relatively simple network, which embedded three modality fusion modules and a fully-connected module, and the satisfactory results demonstrated its ability of recurrence risk stratification.

Computed tomography (CT) and magnetic resonance (MR) have been considered as the routine examinations of cervical cancer patients. Previous studies have suggested that CT and MR images help identify metastatic lymph nodes and distant metastases for patients with cervical cancer (45) and MR images can also evaluate the extent of tumors in the cervix and in the pelvis (46). Additionally, CT and MR images can provide information of tumors, such as lesion size and invasion degree, which is crucial for preliminary clinical staging and prognosis evaluation (47–51). Therefore, many models based on CT or MR images have been proposed for the subtype identification (52), staging analysis (53, 54), lymph node metastasis prediction (54, 55) and prognosis analysis (12, 56, 57) of cervical cancer. Compared to the above methods, the main contributions of this paper lie in the following aspects: (I) We first investigated the feasibility of deep learning method in accurately predicting recurrence risk so as to help formulate the individualized therapeutic schedule for LACC patients. (II) With matched CT and MR images, we proposed a multi-modality model to fully extract modality-specific and modality-sharable features for improving model's performance. (III) We developed a transformer network which can utilize multi-scale and multi-modality discriminative information and experimental results demonstrated its efficacy.

Our study had some limitations. First, our model was constructed only based on imaging (*i.e.*, CT and MR) features, and more integrable factors (e.g., tumor size and tumor marker level) can be collected for further analysis. Second, the VOI segmentation was still a manual process, which was time-consuming and experience-dependent. Last but not the least, this work was a retrospective and single-site study, and a prospective and multi-site cohort is required to further evaluate the model's performance. Nevertheless, to the best of our knowledge, this is the first work to predict the recurrence risk of LACC patients via the deep learning technique, which might supply a valuable reference for the application of deep learning in LACC.

In conclusion, we investigated the ability of transformer network in recurrence risk stratification of LACC based on CT and MR images. The promising results demonstrated that the proposed models might help clinicians make clinical decisions for patients with LACC.

## Data availability statement

The raw data supporting the conclusions of this article will be made available by the authors, without undue reservation.

## Ethics statement

The studies involving human participants were reviewed and approved by the institutional review board of NanFang Hospital, Guangzhou, Guangdong, 510515, PR China. Written informed consent for participation was not required for this study in accordance with the national legislation and the institutional requirements.

## Author contributions

JW: Conceptualization, resources, data curation. YM: Writing - original draft, methodology, visualization. XG: Investigation, resources. YZ: Supervision, funding acquisition, writing - review & editing, conceptualization. All authors contributed to the article and approved the submitted version. We would like to express our sincere gratitude towards Zhenyuan Ning for his treasured and generous support.

## Footnotes

1 Since the data were collected from 2017, we uniformly used the FIGO 2009 staging system instead of the newly revised FIGO 2018.
