# Supplementary material for: Recurrence risk stratification for locally advanced cervical cancer using multi-modality transformer network
Source: Front Oncol. 2023 Feb 16;13:1100087. doi: 10.3389/fonc.2023.1100087 (PMC9978213; doi:10.3389/fonc.2023.1100087)
Supplement: Supplementary file 1 [file DataSheet_1.pdf]

Supplementary Material

1 Architecture of the transformer units

Unlike standard transformer networks, the positional encoding was not included. We first flattened the outputs of spatial pyramid modules (B, H, W, D, C) into sequences (B, L, C) separately, where $L = HWD$ and B, H, W, D, C indicate the batch size, height, width, depth and channel respectively. Then, a linear project layer was used to reduce the complexity of the self-attention mechanism (B, L', C). Afterwards, the self- and cross-attention was employed to capture long-range dependencies of self- and cross-modal features, respectively (shown in Figure S2). The inputs of attention mechanism were a trainable associate memory with a query (Q) vector and a key (K) and value (V) vector pairs, then the output was a feature map of the understanding of V when referring to Q. The formula was as follows:

$$Attention(Q, K, V) = Softmax\left(QK^T / \sqrt{d_k}\right)V$$

where the $\sqrt{d_k}$ term provided appropriate normalization. We first got the augmented representations of self-attention (*i.e.*, S_{CT} and S_{MR}) by mapping Q, K, and V from the same modality, then set the mapping of one modality to Q, and the mapping K, V from the other modality were utilized to obtain the outputs of cross-attention (*i.e.*, C_{CT} and C_{MR}). Finally, the augmented features from self-attention and cross-attention were aggregated with the original features (*i.e.*, X_{CT} and X_{MR}) via a weight average operation:

$$out = \omega_1 S_{CT} + \omega_2 S_{MR} + \omega_3 C_{CT} + \omega_4 C_{MR} + \omega_5 X_{CT} + \omega_6 X_{MR}$$

where the $\{\omega_i\}_{i=1}^{6}$ are learned by the network and their initial values were all 1/6. Subsequently, the output passed through a MLP layer and a complete vision transformer module in sequence to deepen the understanding of filtered features. After that, the feature sequences were reshaped into original size, and then connected with the outputs of spatial pyramid modules in the channel dimension.

About the parameters of transformer units in the three modal fusion modules, $C = \{4, 8, 16\}$, $L' = \{32, 16, 8\}$, $\beta = 2$ and $d_k = \{16, 8, 4\}$. The batch size and epoch number are set to 135 and 200, respectively. Detailed configuration of the network can be seen in Figure S3.

2 Efficacy of multi-modality data

2.1 Experimental design

In order to make a comparison between multi-modality and mono-modality data, we fine-tuned the transformer network to make training and testing also possible for mono-modality data. Specifically, we modified the transformer units to output the feature maps only from the multi-head self-attention mechanism. Besides, the final output was generated after going through the Sigmoid activation.

3 Efficacy of key modules in transformer network

3.1 Experimental design

To verify the efficacy of key modules in transformer network, we conducted some variant experiments: (I) each transformer unit was replaced by two consecutive convolutional layers with kernel sizes of $3 \times 3 \times 3$ (denoted as *proposed_woTF*). (II) two $3 \times 3 \times 3$ convolutional layers were utilized to substitute the spatial pyramid unit (denoted as *proposed_woSP*) to explore the better feature extractor. (III) the *weight average* operator was replaced by *average* or *concatenation* in the modality fusion module and decision-making stage, respectively. (IV) the number of the modality fusion module was increased to four or decreased to two to reflect the suitability of models.

3.2 Results

Table S1 and Table S2 show quantitative comparisons of transformer networks with *proposed_woTF* and *proposed_woSP* models, respectively. The proposed models have clear advantages for recurrence risk stratification of LACC on both multi-modality and mono-modality data. We can conclude that, the transformer unit can fuse long-range dependence and the spatial pyramid module is helpful to fuse multi-scale information with larger receptive field. Therefore, they can both boost performances.

The ablation experience of weight average operator is posted in Table S3. The average does not have advantages in the modality fusion module and decision-making stage, because it cannot adaptively combine information, while the concatenation has closer performance to weight average in modality fusion module because the subsequent multi-layer perceptron can implicitly learn more efficient feature representations. While, in the decision-making stage, the concatenation does not restrict the sum of the weights of the two modalities, which will make the value of fusion prediction probability become larger or smaller. In the contrast, the weight average has an absolute advantage, with AUC and accuracy both improving by approximately 10% compared to others.

In the ablation experiments of model depth (Table S4), the model with three modality fusion modules obtained the best results when compared with the models with two or four. Consequently, we intuitively think that three modality fusion modules are the optimal configuration for current dataset and networks.

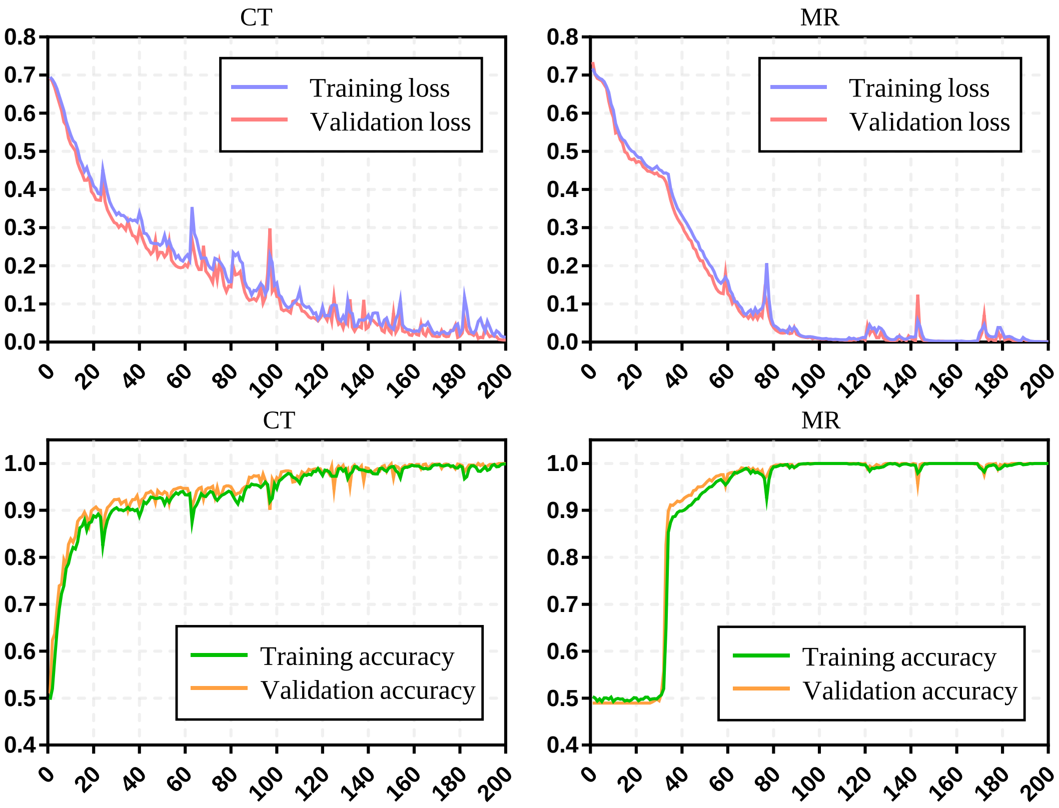

Figure S1. The training process of transformer network on mono-modality data.

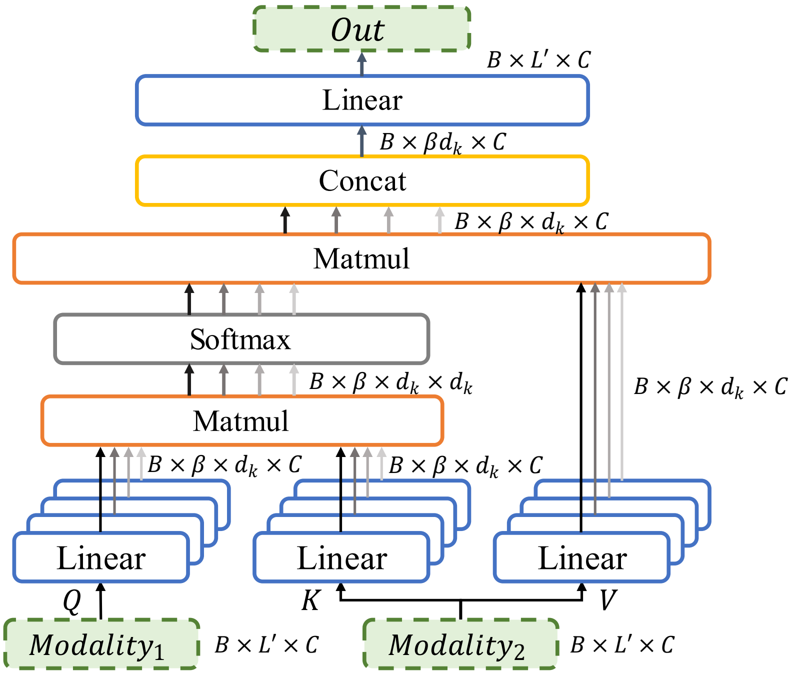

Figure S2. The mechanism of attention.

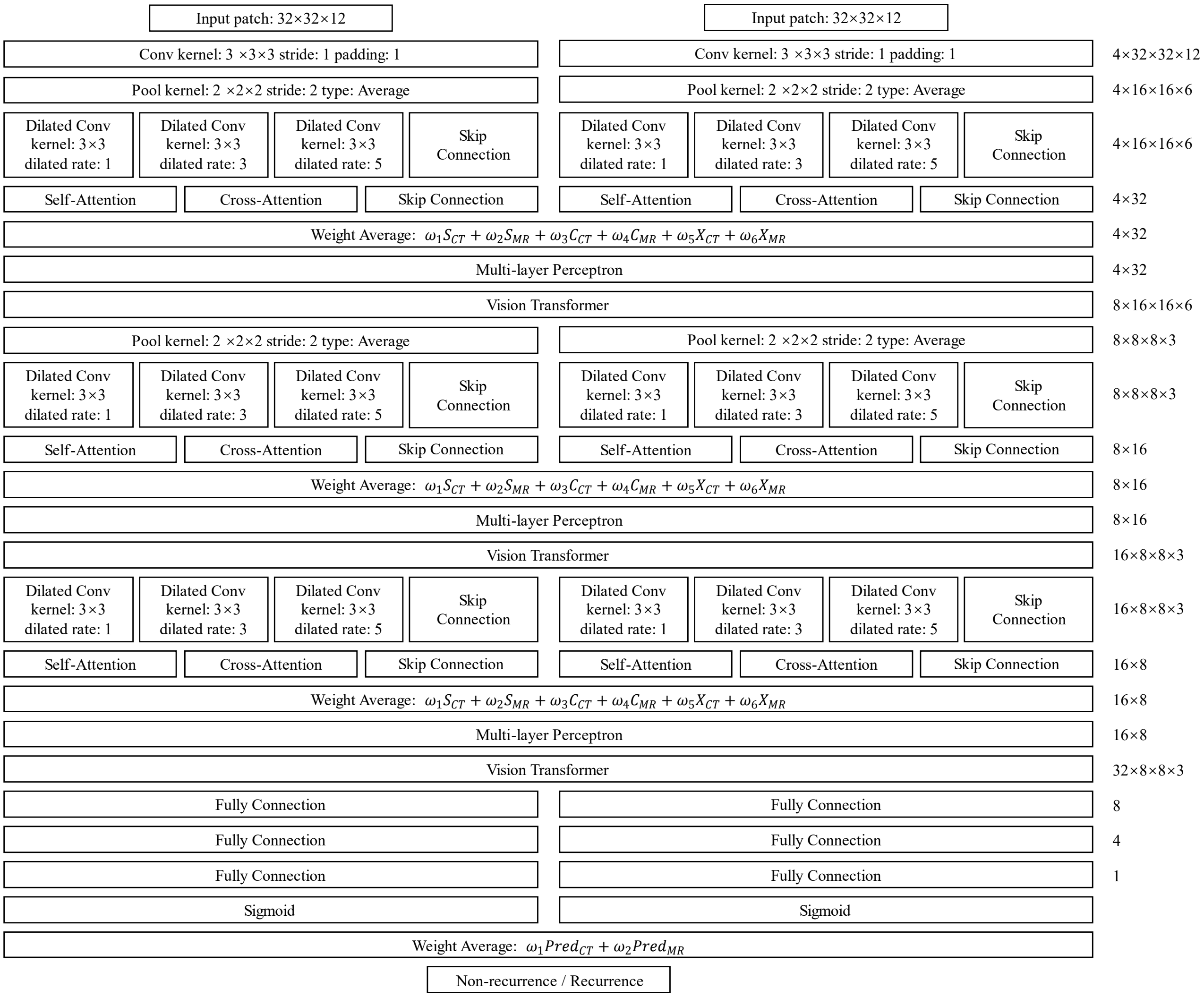

Figure S3. Detailed configuration of the network

Table S1. The qualitative results of proposed model with and without the transformer units.

Modality	Modality	AUC	Accuracy	F1-score	Sensitivity	Specificity	Precision
Proposed	CT + MR	0.819±0.038	0.869±0.023	0.914±0.016	0.911±0.038	0.725±0.094	0.919±0.025
	CT	0.677±0.021	0.737±0.042	0.813±0.039	0.748±0.068	0.700±0.061	0.895±0.013
	MR	0.676±0.068	0.720±0.066	0.795±0.049	0.704±0.052	0.775±0.146	0.914±0.055
Proposed_woTF	CT + MR	0.731±0.037	0.800±0.031	0.865±0.025	0.837±0.055	0.675±0.061	0.898±0.013
	CT	0.727±0.017	0.783±0.039	0.846±0.040	0.793±0.090	0.750±0.137	0.921±0.042
	MR	0.710±0.037	0.783±0.029	0.852±0.020	0.807±0.028	0.700±0.100	0.902±0.031

Table S2. Performance comparison with and without the spatial pyramid unit in each modal fusion module.

Method	Modality	AUC	Accuracy	F1-score	Sensitivity	Specificity	Precision
Proposed	CT + MR	0.819±0.038	0.869±0.023	0.914±0.016	0.911±0.038	0.725±0.094	0.919±0.025
	CT	0.677±0.021	0.737±0.042	0.813±0.039	0.748±0.068	0.700±0.061	0.895±0.013
	MR	0.676±0.068	0.720±0.066	0.795±0.049	0.704±0.052	0.775±0.146	0.914±0.055
Proposed_woSP	CT + MR	0.787±0.016	0.840±0.014	0.888±0.013	0.830±0.050	0.875±0.137	0.961±0.039
	CT	0.749±0.039	0.806±0.049	0.866±0.044	0.830±0.090	0.725±0.122	0.914±0.031
	MR	0.655±0.016	0.691±0.021	0.770±0.023	0.674±0.043	0.750±0.079	0.903±0.024

Table S3. *weight average* vs. *average* and *concatenation*.

Modality fusion module	Decision making stage	AUC	Accuracy	F1-score	Sensitivity	Specificity	Precision
Weight average	Weight average	0.819±0.038	0.869±0.023	0.914±0.016	0.911±0.038	0.725±0.094	0.919±0.025
Average	Weight average	0.724±0.005	0.800±0.000	0.866±0.003	0.837±0.018	0.675±0.061	0.897±0.016
Concatenate	Weight average	0.773±0.024	0.834±0.021	0.887±0.017	0.844±0.043	0.800±0.127	0.937±0.038
Weight average	Average	0.714±0.012	0.794±0.011	0.864±0.009	0.844±0.015	0.625±0.000	0.884±0.002
Weight average	Concatenate	0.685±0.013	0.749±0.011	0.823±0.009	0.756±0.018	0.725±0.05	0.903±0.014

Table S4. Ablation experiments to explore the optimal depth of the model. Number represents the amount of the modality fusion module.

Number	Modality	AUC	Accuracy	F1-score	Sensitivity	Specificity	Precision
Two	CT + MR	0.715±0.007	0.766±0.033	0.831±0.037	0.763±0.086	0.775±0.146	0.926±0.044
	CT	0.707±0.033	0.783±0.029	0.853±0.022	0.822±0.036	0.650±0.050	0.888±0.015
	MR	0.639±0.010	0.674±0.014	0.757±0.015	0.659±0.028	0.725±0.050	0.891±0.014
Three	CT + MR	0.819±0.038	0.869±0.023	0.914±0.016	0.911±0.038	0.725±0.094	0.919±0.025
	CT	0.677±0.021	0.737±0.042	0.813±0.039	0.748±0.068	0.700±0.061	0.895±0.013
	MR	0.676±0.068	0.720±0.066	0.795±0.049	0.704±0.052	0.775±0.146	0.914±0.055
Four	CT + MR	0.782±0.020	0.846±0.014	0.899±0.010	0.889±0.023	0.700±0.061	0.910±0.015
	CT	0.720±0.008	0.794±0.011	0.861±0.011	0.830±0.030	0.675±0.061	0.897±0.015
	MR	0.721±0.019	0.771±0.031	0.836±0.029	0.763±0.069	0.800±0.100	0.931±0.026
